# Systematic Review of Pharmacist-Administered Paediatric Vaccinations in Low- and Lower-Middle-Income Countries

**DOI:** 10.3390/vaccines14080661

**Published:** 2026-07-28

**Authors:** Kimberly McKeirnan, Ilse Truter, Teri-Lynne Fogarty

**Affiliations:** 1 Department of Pharmacy, Nelson Mandela University, Gqeberha 6031, South Africa; ilse.truter@mandela.ac.za (I.T.); teri-lynne.fogarty@mandela.ac.za (T.-L.F.); 2 Department of Pharmacotherapy, Washington State University, Spokane, WA 99202, USA

**Keywords:** pharmacy-based vaccination, pharmacist-administered immunisation, paediatric, low- and lower-middle-income countries

## Abstract

Background: An estimated 25 million children globally miss routine vaccinations each year, with 14.3 million classified as zero-dose and the majority residing in low- and lower-middle-income countries (LMICs). Pharmacies are recognised as accessible healthcare delivery points, yet their role in paediatric immunisation in LMICs is not well defined. This literature review sought to evaluate pharmacist-provided vaccination services for paediatric patients in LMICs. Methods: A systematic review was conducted following Cochrane and PRISMA guidelines. Five databases and grey literature were searched for articles published between January 2005 and June 2026. Studies were reviewed by multiple authors to reduce the risk of individual bias and included if they reported original research involving pharmacists in LMICs delivering vaccination-related services to children aged 12 years and under. For each study, items intended for extraction included country, pharmacy setting, specific vaccines administered, patient age range, and specific roles for the pharmacist in the vaccination administration process. The protocol for this systematic review was registered with the Nelson Mandela University Faculty of Health Sciences Postgraduate Studies Committee and the Research Ethics Committee for Humans (H23-HEA-PHA-007). Results: Of 162 identified records, four studies from Bangladesh, Ethiopia, India, and Jordan met inclusion criteria. No study described pharmacists routinely administering paediatric vaccines. Instead, findings focused on indirect involvement, system readiness, and public perception. Significant barriers included limited infrastructure, inadequate training, and lack of regulatory authority. Study results were limited by a lack of information published and inclusion of only articles available in English. Conclusions: Evidence on pharmacist-administered paediatric vaccinations in LMICs is scarce. Expanding pharmacist roles could improve access and reduce zero-dose prevalence, but would require policy support, infrastructure investment, and further implementation research.

## 1. Introduction

Vaccines are among the most life-saving health interventions in human history and prevent an estimated 3.5–5 million deaths globally each year [1,2]. However, the United Nations Children’s Fund (UNICEF) estimates that 25 million children globally miss routine vaccines annually [3]. Of those, the World Health Organization (WHO) reports that 14.3 million are considered zero-dose children [4], meaning that they have not received a single dose of any vaccine [5]. Zero-dose children account for nearly half of all vaccine-preventable deaths [5]. The majority (87%) of zero-dose children live in low- and lower-middle-income countries (LMICs) [6]. The WHO and UNICEF Estimates of National Immunization Coverage (WUENIC) estimates show that half of all zero-dose children live in just six large-population LMICs (India, Nigeria, Indonesia, Ethiopia, the Philippines, and the Democratic Republic of the Congo) [6].

Public health barriers that lead to the existence of zero-dose children are numerous, and their presence is often an indicator of health inequities [7]. Poverty is a strong predictor. The majority of families with zero-dose children subsist on less than the poverty line equivalent of $1.90 per day [8]. Other reported barriers to vaccination for zero-dose children include ethnicity [9], religion [10], gender-related barriers [11], nomadic or migrant lifestyles [12], and displacement due to war, conflict, food shortages, and/or natural disasters [12].

To address concerning trends related to zero-dose children, the WHO’s Immunization Agenda 2030 includes a strategic priority for the integration of life course vaccination in all member states [13]. This strategic priority includes the goal of establishing “integrated delivery touchpoints” for vaccinations administered to patients of all ages. Pharmacies have repeatedly proven to be effective integrated delivery touchpoints, providing accessible points of care for patients globally. A 2023 review by Romero-Mancilla and colleagues highlighted benefits resulting from vaccination services in community pharmacies, including increasing vaccine accessibility and decreasing the impact of vaccine-related health disparities, particularly for patients in underserved areas [14].

Most successful examples of the impact of vaccinations given in pharmacies come from high-income countries (HICs) and involve adult patients [14]. In the US, pharmacists have administered more vaccines than any other provider type since 2020 [15]. In Portugal, approximately one-third of vaccinations are administered by pharmacists [16]. In Australia, 47% of COVID-19 vaccinations were administered by pharmacists [17]. Many other HICs, including the UK, France, and Saudi Arabia, demonstrate the success of pharmacy-based vaccinations [14].

In LMICs, regulations governing vaccine delivery vary, with differences in pharmacists’ scope of practice, authorization to administer vaccines, requirements for immunisation training, access to patient health records, and policies regarding which vaccination services, if any, can be provided [18,19]. These regulatory differences influence the extent to which pharmacists can participate in vaccination services. When pharmacists are allowed to be involved, results demonstrate a positive impact on public health in some LMICs [18]. A 2021 review by Yemeke and colleagues focused on community pharmacy vaccinations in LMICs showed that pharmacists contributed to improved vaccine uptake by providing convenient care, being trusted healthcare providers, and providing vaccine education and advocacy [18].

Recent literature reviews have evaluated pharmacy-based immunisation services globally [14] and specifically the role of pharmacists as immunisers in LMICs [18]. However, limited information is available about how pharmacists are involved in the vaccination of paediatric patients in LMICs. Several recent commentaries have identified opportunities for pharmacists to be involved in vaccinating children [20], vaccine safety monitoring [21], encouraging public awareness [22], and identifying children who have missed routine immunisations [23]. Given the potential impact pharmacists can have on paediatric vaccination rates and zero-dose children, further evaluation of pharmacy-based vaccination efforts for children is warranted. The objective of this work was to evaluate pharmacist-provided vaccination services for paediatric patients in LMICs.

## 2. Materials and Methods

### 2.1. Study Design

A systematic literature review was conducted following the methods described in the Cochrane Handbook for Systematic Reviews [24] and the Preferred Reporting Items for Systematic Reviews and Meta-Analyses (PRISMA) reporting guidelines [25]. The protocol for this systematic review was registered with the Nelson Mandela University Faculty of Health Sciences Postgraduate Studies Committee and the Research Ethics Committee for Humans (H23-HEA-PHA-007).

### 2.2. Search Strategy

A literature search was conducted using PubMed, the Cochrane Central Register of Controlled Trials (CENTRAL), the Cumulative Index to Nursing and Allied Health Literature (CINAHL), Web of Science, and Embase on 18 June 2026. The search strategy, organized by website, is included as Appendix A. Each search included four term groupings:

- Group 1: Variations of the terms pharmacist and pharmacy used in different global regions (i.e., druggist, chemist, and dispensary, retail drug shop).
- Group 2: Variations of the terms vaccinate and immunise using both British and American spellings (i.e., vaccinator, immunizer, immuniser).
- Group 3: Variations of terms related to paediatrics using both British and American spellings (i.e., child, childhood, pediatric, paediatric) and terms related to paediatric vaccinations, such as “routine” or “life course”.
- Group 4: Names of individual LMICs from the World Bank country classification list for low- or low-middle-income countries [26].

Medical Subject Heading (MeSH) terms and term abbreviations (i.e., “immuniz* or pharmac*) were utilised where available. A search for grey literature, defined by Cochrane as “reports published outside of traditional commercial publishing” was conducted using Google and combinations of the study search terms from Appendix A to reduce the risk of publication bias and to identify as many relevant articles as possible [24]. A manual search of the references of the retrieved articles and the references from two recent global systematic literature reviews about pharmacists vaccinating [14,18] was also conducted. A Faculty of Health Sciences research librarian was also consulted to verify the completeness of the search strategy and to determine whether additional databases should be included.

### 2.3. Eligibility Criteria

Relevant articles were then screened using the following eligibility criteria:

- All articles published in English or with an available online English translation that were published between 1 January 2005 and the date of the search were included.
- Articles must have included original research. Commentaries, literature reviews, and conference abstracts were not included.
- Studies must have described activities in LMICs [26].
- Pharmacy personnel, including pharmacists, pharmacy students, or members of the pharmacy support workforce (PSW) [27], must have been directly involved in the vaccination process. Articles describing vaccination services offered in pharmacies but administered by other types of healthcare providers, most commonly nurses, without pharmacy personnel involvement were excluded. The role of the pharmacy personnel could vary, including screening, prescribing, counselling, educating, and administering the vaccine, provided that pharmacy personnel were involved in a direct patient care capacity.
- Articles must have included vaccination services for paediatric patients aged 12 years or younger. Articles describing pharmacy vaccination services for adolescents and adults were excluded if paediatric patients were not also mentioned.

### 2.4. Study Selection

After duplicates were removed, article titles were reviewed for relevance. The abstracts of the remaining articles were retrieved and reviewed by all three authors independently using the eligibility criteria. Full-text articles were retrieved for all articles meeting eligibility criteria and evaluated independently by all three authors. Disagreements were resolved through group discussion and review of the eligibility criteria. Automation tools were not used.

### 2.5. Data Extraction

Prespecified data were extracted by one author (KM) and reviewed by the other two authors (IT, TF) to reduce individual bias. For each study, items intended for extraction included country, pharmacy setting, specific vaccines administered, patient age range, and specific roles for the pharmacist in the vaccination administration process.

## 3. Results

**Figure 1:**
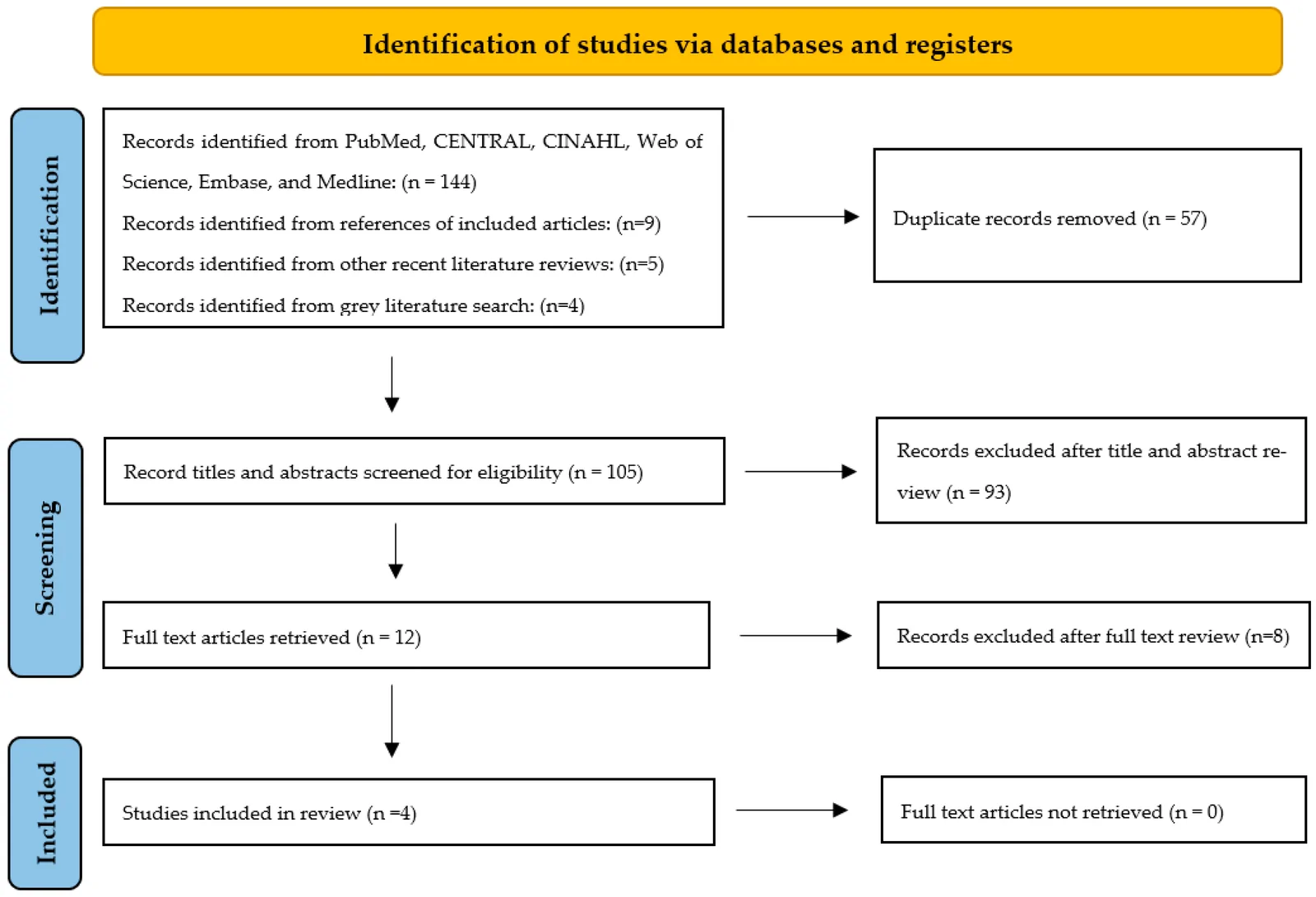
PRISMA flow diagram for inclusion of studies in *A Systematic Review of Pharmacist-Administered Paediatric Vaccinations in Low- and Low-Middle-Income Countries* [24]. Abbreviations used: CENTRAL: Cochrane Central Register of Controlled Trials; CINAHL: Cumulative Index to Nursing and Allied Health Literature.

A total of 162 articles were identified using the search strategy. After removing 57 duplicated articles, the titles and abstracts of 105 articles were screened for eligibility. Of those, 93 were excluded, and the full text of 12 articles was reviewed. After inclusion criteria were applied, a total of four studies [28,29,30,31] from four different countries were included, representing Bangladesh, Ethiopia, India, and Jordan, as shown in the PRISMA flow diagram (Figure 1). Reviewer agreement for article inclusion was assessed using Fleiss’ kappa and yielded a coefficient of 0.77, indicating substantial agreement [32]. A list of the full text studies reviewed but excluded [20,21,23,33,34,35,36,37], with reasons for exclusion in shown in Appendix B. All four included articles described the involvement of pharmacists in the vaccination of children. A summary of the included articles is shown in Table 1.

The review was intended to evaluate the implementation of paediatric vaccination services in pharmacies in LMICs); however, no detailed accounts of paediatric vaccination services were identified. One of the four studies was designed to determine whether any pharmacies in a geographic region were administering vaccines to children, but few details were provided [29]. Another gathered public opinion about pharmacists as immunisers, including community members’ views of paediatric vaccinations, but did not provide details about the services themselves [30]. One evaluated the readiness of healthcare facilities, including pharmacies, to offer paediatric vaccination services [31]. The fourth article described a randomised controlled trial that involved pharmacists in the vaccination process, but as educators rather than injectors [28]. Although none of these articles offered a detailed look into paediatric vaccination practices in pharmacies, they did provide insight into the challenges and opportunities of pharmacy practice in LMICs.

### 3.1. Readiness of LMIC Pharmacists for Paediatric Vaccination Administration

Two of the articles evaluated different aspects of pharmacist readiness to either administer vaccinations or be involved in the vaccination process. A study by Arvind and colleagues in India conducted a randomised controlled trial to evaluate the impact of a clinical pharmacist on reducing adverse events following immunisation [28]. Although pharmacists in the study did not administer vaccinations, they provided education and information about vaccinations directly to the patients being vaccinated. The authors explained that the decision to vaccinate their children is always the choice of the parent, but that pharmacists can provide instrumental information about the risks and benefits of vaccinations to aid them in making the decision [28]. Arvind and colleagues described the pharmacist’s role in providing vaccination education for parents and reducing adverse events following immunisation (AEFI). At the time of this writing, pharmacists in India were not authorised to vaccinate, although advocacy efforts by the India Pharmacists Association [38] and press releases from the Times of India [39] reported that 1.2 million pharmacists were being trained to vaccinate.

Similarly to the study by Arvind and colleagues, Batarseh and colleagues did not describe the implementation of vaccinations administered by pharmacists but rather evaluated the opportunity to take on this role [30]. Batarseh and colleagues evaluated public perceptions regarding pharmacists as immunisers in Jordan. When the study was published in 2021, pharmacists were not immunisers [30], although the role was beginning to expand. Batarseh and colleagues conducted surveys with members of the public via telephone to assess public perceptions of pharmacists as immunisers in Jordan. Results showed that the public generally had a positive opinion about pharmacists administering vaccinations. The majority also agreed they would be willing to allow a pharmacist to vaccinate their children. Like Arvind and colleagues, Batarseh and colleagues utilised the results of their study to advocate for expanding pharmacist roles to include vaccine administration [28,30]. At the time of publication in 2021, Jordanian pharmacists were not routinely administering immunisations, but the Jordanian Pharmacist Association had launched campaigns designed to evaluate need and opportunity [40]. Batarseh and colleagues also suggested that pharmacists could build on strong existing relations and accessibility in their local community to offer vaccination services, which has been done in many other countries globally [30].

### 3.2. Readiness of LMIC Pharmacies to Provide Paediatric Vaccinations

The studies from Bangladesh and Ethiopia evaluated the preparedness of pharmacies, rather than pharmacists, for paediatric vaccine administration. Shawon and colleagues evaluated the readiness of healthcare facilities to offer vaccination services using the Service Availability and Readiness Assessment manual created by the WHO [31,41]. Results showed that a lack of vaccine storage capabilities, sufficient funding and resources, and vaccination training programmes were barriers to administering vaccinations in pharmacies. Of the 12 pharmacies evaluated in the study, none offered vaccination services. Only four (33%) had reliable electricity and only one (8.3%) had a consultation space and adequate sanitation facilities for patients. Since many study pharmacies lacked the resources the WHO considers “basic amenities”, pharmacists in these locations were unlikely to be positioned to offer vaccination services [31].

Ayele and colleagues conducted a survey of pharmacists in 238 community retail drug outlets in the Amhara regional state of Ethiopia [29]. The survey evaluated the pharmacists’ provision of several medical and health services for children. Vaccination had the lowest mean involvement score compared to other services, which included discussing vitamins and supplements, answering questions about minor health problems like fever and diarrhoea, and providing advice about infant formula and milk [29].

### 3.3. Paediatric Vaccinations Provided in LMICs

Studies included in this review were also evaluated to determine which vaccines were being provided to paediatric patients in LMICs. Results are shown in Table 2.

## 4. Discussion

### 4.1. Limited Evidence

This review sought to evaluate pharmacist-provided paediatric vaccination services in LMICs. Despite an extensive search, only four studies met the inclusion criteria, and none provided detailed descriptions of pharmacists actively involved in administering paediatric vaccines in practice. This finding highlights a substantial gap in literature and suggests that, although pharmacists are increasingly recognised as accessible healthcare providers who offer important services to their communities, their role in paediatric vaccinations remains underdeveloped, underreported, or both. With 80 countries classified as low- or lower-middle-income countries [26], the opportunity to increase global paediatric vaccination coverage abounds.

The most prominent finding from this review is the scarcity of empirical evidence describing pharmacy-based paediatric vaccination services in LMICs. While pharmacists have repeatedly demonstrated significant contributions to vaccination delivery in HICs and to adult vaccinations in LMICs [14], models for vaccinating children in LMICs appear to be in early stages of development. Notably, not all countries that embrace pharmacists as vaccinators of children are HICs. A previous review by Meyer and colleagues showed that pharmacists have an important role to play in childhood vaccinations and were actively involved in South Africa even prior to COVID-19 [42]. The studies included in the current review primarily focused on perceptions, readiness, or indirect roles rather than implementation of services. This discordance between recognised potential and documented practice underscores an important research and practice gap.

### 4.2. Evolving Roles for Pharmacists

Findings from Arvid and colleagues suggest that pharmacists are well-positioned to expand their roles in paediatric vaccination. In India, pharmacists contributed meaningfully to improving caregivers’ knowledge, attitudes, and practices regarding vaccination through counselling and education. Although pharmacists in the study did not administer vaccines, their involvement demonstrates the value of integrating pharmacists into the vaccination continuum of care. Even when pharmacists are not allowed to administer vaccinations, they can still have a positive impact on the community through patient education and outreach. In 2026, Agha and colleagues described pharmacists as social media influencers who encouraged adolescent girls to be vaccinated against Human Papillomavirus (HPV) in Nigeria. Through social media outreach, pharmacists provided evidenced-based information about the HPV vaccine to girls and caregivers. Results showed that adolescent girls reached by the campaign were 1.48 times more likely to be vaccinated than peers elsewhere [43].

Similarly, in Jordan, public perception data from Batarseh and colleagues indicated strong acceptance of pharmacists as immunisers, including for paediatric populations. Public trust is a critical determinant of successful vaccine delivery, and these findings suggest that pharmacists could serve as effective vaccination providers if regulatory frameworks evolve to support this role. Other studies have also shown readiness among Jordanian pharmacists to vaccinate [44,45] as long as proper training and infrastructure are available. While paediatric vaccination by pharmacists is not yet routine, these developments suggest a growing interest and acceptance that could facilitate expansion to children in the future. Continued professional advocacy, regulatory reform, and targeted training programmes will be essential to support this transition.

The limited number of studies identified in this review reflects both structural and regulatory barriers that restrict pharmacists’ scope of practice in many LMICs. In the included studies, pharmacists were not yet authorised to administer vaccines, which likely contributed to the absence of implementation-focused research. Additionally, the underrepresentation of paediatric services specifically suggests that, even where adult vaccination programmes exist, extension to children may face additional regulatory, clinical, and societal barriers. Additional literature that did not meet the inclusion criteria highlighted the same issues in other LMICs. Mayora and colleagues surveyed private retail drug shops in rural Uganda and identified that many (65%) offered care services for paediatric patients and some (12%) offered vaccination services, although no specific information about paediatric vaccinations was mentioned [46]. However, the same study also reported that the Ugandan National Drug Authority guidelines and policy framework does not allow retail drug shops to provide vaccinations [46]. Similarly, Aelita and colleagues advocated for Ukrainian pharmaceutical workers to have vaccination of children included as part of their professional responsibilities [47]. Chukhray and colleagues described how allowing Ukrainian pharmacists to vaccinate could be valuable, but would require regulatory support, training, additional pharmacy staff, a system for record-keeping and reimbursement, and equipment [34].

### 4.3. System-Level Barriers

In contrast to pharmacist willingness and capability, studies from Bangladesh and Ethiopia highlight significant system-level barriers that limit the implementation of pharmacy-based paediatric vaccination services. In Bangladesh, Shawon and colleagues described inadequate infrastructure, including a lack of cold chain capacity, unreliable electricity, insufficient sanitation facilities, and limited consultation space as major limiting factors [31]. These findings emphasise that the successful implementation of vaccination services requires not only trained personnel but also adequate physical and logistical resources. Similarly, in Ethiopia, Ayele and colleagues reported that pharmacists have minimal involvement in vaccination services compared to other child health services [29]. This suggests that even where pharmacists are engaged in paediatric care, vaccination activities may not be prioritised or supported. Competing responsibilities and the absence of integration with national vaccination programmes may contribute to this trend. Two recent literature reviews also identified that a lack of available vaccination training opportunities [48,49] may also limit the ability of pharmacists to be involved in vaccination services.

During the literature search, several studies were identified that did not meet inclusion criteria but still provided important insight into system-level barriers for pharmacists in LMICs and were aligned with the results of the studies by Ayele and Shawon included in this review. In Nigeria, Oladigbolu and colleagues reported that most pharmacists surveyed (91%) were willing to vaccinate, but that inadequate funding, equipment, and training were reported as barriers [50]. Aderemi-Williams and Igwilo reported similar results from Nigeria: pharmacies could be a feasible vaccination location, but infrastructure including refrigerators, freezers, and generators would be needed [33].

Another opportunity exists in involving the PSW [27]. Although pharmacy technicians are an important part of pharmacy-based vaccination services in many HICs [51] for patients of all ages [52], no studies were identified that described their involvement in LMICs. Identifying opportunities to train [53] and engage the PSW could reduce the level of effort needed by pharmacists and increase the number of trained vaccinators available in pharmacies [54].

### 4.4. Opportunities for Expanding Pharmacist Involvement

Despite these challenges, several opportunities exist to strengthen the role of pharmacists in paediatric vaccinations in LMICs. First, pharmacists are among the most accessible healthcare providers, particularly in underserved and rural areas where zero-dose children are often concentrated. Leveraging pharmacies as “integrated delivery touchpoints,” as emphasised in Immunization Agenda 2030 [13], could improve vaccine access and reduce disparities.

Second, pharmacists can play critical roles beyond vaccine administration such as providing caregiver education, addressing vaccine hesitancy, and supporting adverse event monitoring. Even in settings where regulatory restrictions limit their ability to administer vaccines, these contributions can meaningfully improve immunisation coverage.

Third, targeted investments in infrastructure and training could enable pharmacies to meet minimum standards for vaccine storage and delivery. Partnerships between governments, professional organisations, and international agencies may help address resource gaps and integrate pharmacies into national immunisation systems.

### 4.5. Limitations, Strengths, and Next Steps

This review has several strengths, including adherence to Cochrane methodology and PRISMA guidelines, a comprehensive and systematic search strategy, and the inclusion of multiple databases and grey literature. However, the findings must be interpreted within the context of the study’s limitations.

This review evaluated published literature describing pharmacists as part of the vaccination process for children in LMICs. It is likely that there are ongoing efforts by LMIC pharmacists that are not documented in published literature. Peer-reviewed publications are disproportionately produced by academic institutions and well-resourced health systems, which are concentrated in high-income countries, while LMICs face constraints including limited research infrastructure, funding, and academic training capacity [55]. Pharmacists serving LMIC populations may be offering paediatric vaccination services that are not documented in published literature.

The search strategy for this review used the names of individual LMIC countries. The authors attempted several search combinations and found this method most effective at identifying LMIC countries. However, the authors acknowledge that this approach may have unintentionally excluded multi-country studies or those summarizing global vaccination data. A thorough grey literature search was also conducted to minimize this risk.

This review included only studies published in English or those available with an English translation. As many LMICs do not use English as a national language, it is possible that relevant studies were not identified due to language limitations in the search strategy. Consequently, important evidence published in other languages may have been excluded. Future research should consider incorporating non-English sources to provide a more comprehensive understanding of this topic.

Given the limited evidence identified, there is a clear need for further research. Future studies describing pilot programmes and the implementation of pharmacist-administered paediatric vaccinations in LMIC settings would be a useful next step. Comparative studies assessing outcomes such as vaccine uptake, cost-effectiveness, and patient satisfaction would be particularly valuable. Additionally, many studies describe issues with advocacy and legal authority to administer vaccines. Policy analyses examining regulatory frameworks and their impact on pharmacists’ scope of practice could help inform strategies for expanding services. An evaluation of regulatory policies in African countries is currently underway.

## 5. Conclusions

Few publications exist describing paediatric vaccination services provided by pharmacists in LMICs despite a substantial need for a larger vaccination workforce in those countries. Pharmacies have repeatedly proven themselves as effective integrated delivery touchpoints in many HICs, providing accessible points of care for patients globally. Expanding pharmacist vaccination services in LMICs could be a first step to increase the number of available vaccinators, reduce low paediatric vaccination rates, and reduce the number of zero-dose children worldwide.

## Figures and Tables

**Table 1:** Articles identified for inclusion in *Systematic Review of Pharmacist-Administered Paediatric Vaccinations in Low- and Low-Middle-Income Countries*.

Authors, Year	Title	Journal	Study Design, Study Objectives	Country, Sample Size, Participant Characteristics	Relevant Key Findings	Study Limitations
Arvind and Colleagues, 2022 [28]	A Randomized, Single Centered, Parallel and Open Labelled Interventional Study on Effectiveness of Clinical Pharmacists on Adverse Event Following Immunization (AEFI) in Pediatric Population	Current Drug Safety	Randomised controlled trial, To assess the effectiveness of clinical pharmacist intervention on an adverse event following a pediatric immunization (AEFI)	India, n = 88 parents/guardians of patients less than 5 years of age at a tertiary care hospital	Involving pharmacists in India in the immunisation process to provide patient counselling improved paediatric patient guardians’ knowledge, attitudes, and practices regarding immunisations.	Small sample size; low frequency of occurrence of individual AEFIs; conducted at one hospital
Ayele and Colleagues, 2022 [29]	Role of community pharmacy professionals in child health service provision in Ethiopia: a cross-sectional survey in six cities of Amhara regional state	BMC Health Services Research	Cross-sectional survey, To evaluate the level of involvement of community pharmacy professionals in child health services	Ethiopia, n = 238 community pharmacists in the Amhara region	Community pharmacists in Ethiopia provide childhood vaccination services but are more frequently involved in providing other child health services.	Self-reported responses; conducted in one region (Amhara) of Ethiopia
Batarseh and Colleagues, 2021 [30]	Perception and attitude of the public on vaccine practices and pharmacists as immunizers in Jordan	Journal of Pharmaceutical Health Service Research	Cross-sectional survey, To assess general perception and attitude of the public on vaccines current practices and pharmacists as immunizers	Jordan, n = 366 random sampling of people living in Amman, Irbid, and Zarqa	The Jordanian public generally had a positive opinion about pharmacists administering vaccinations and the majority were willing to allow a pharmacist to vaccinate their children.	Low response rate among targeted population
Shawon and Colleagues, 2018 [31]	General service and child immunization-specific readiness assessment of healthcare facilities in two select divisions in Bangladesh	BMC Health Services Research	Survey, To assess general service and child immunization-specific healthcare facility readiness to provide vaccination services	Bangladesh, n = 123 healthcare facilities, (61 from Rajshahi and 62 from Sylhet)	A lack of vaccine storage capabilities, sufficient funding and resources, and vaccination training programmes were barriers to administering vaccinations in Bangladesh pharmacies.	Healthcare facilities were from purposefully selected regions; small sample size

**Table 2:** Vaccines administered in included studies.

Author and Year	Vaccines Included in Study
Arvind and colleagues 2022 [28]	BCG, DPT, IPV, OPV, MMR and MMR booster, Hepatitis B, PVC, rotavirus, Penta 3 *
Ayele and colleagues 2022 [29]	Information not provided
Batarseh and colleagues 2021 [30]	Information not provided
Shawon and colleagues 2018 [31]	MMR, Penta 3, IPV, BCG, PCV

Abbreviation used: BCG: Bacillus Calmette-Guerin; DPT: diphtheria, pertussis, tetanus; MMR: measles, mumps rubella; IPV: inactivated polio vaccine; OPV: oral polio vaccine; PCV: pneumococcal conjugate vaccine. * Penta 3 contains vaccines for diphtheria, tetanus, acellular pertussis, inactivated polio virus, and Haemophilus influenza type b.

## Data Availability

The datasets presented in this article are not readily available because of restrictions from the Research Ethics Committee (Human). Requests to access the datasets should be directed to the corresponding author.

## Abbreviations

The following abbreviations are used in this manuscript:

BCG	Bacillus Calmette-Guerin
CENTRAL	Cochrane Central Register of Controlled Trials
CINAHL	Cumulative Index to Nursing and Allied Health Literature.
DPT	Diphtheria, pertussis, tetanus
IPV	Inactivated polio virus
LMIC	Low- and low-middle-income countries
MMR	Measles, mumps, rubella
OPV	Oral polio vaccine
PCV	Pneumococcal conjugate vaccine

## Appendix A. Search Strategy

### Appendix A.1. Pubmed (n = 45)

(((pharmacy[tiab] OR pharmacies[tiab] OR pharmacist[tiab] OR pharmacists[tiab] OR chemist[tiab] OR chemists[tiab] OR druggist[tiab] OR druggists[tiab]) AND (vaccine[tiab] OR vaccinat*[tiab] OR immuniz*[tiab] OR immunis*[tiab] or inocula*[tiab])) AND (Afghanistan[tiab] OR Angola[tiab] OR Burundi[tiab] OR Benin[tiab] OR “Burkina Faso”[tiab] OR Bangladesh[tiab] OR Bolivia[tiab] OR Bhutan[tiab] OR “Central African Republic”[tiab] OR “Côte d’Ivoire” [tiab] OR Cameroon[tiab] OR “Democratic Republic of Congo” [tiab] OR Congo[tiab] OR Comoros[tiab] OR “Cabo Verde” [tiab] OR Djibouti[tiab] OR Algeria[tiab] OR Egypt[tiab] OR Eritrea[tiab] OR Ethiopia[tiab] OR Micronesia[tiab] OR Ghana[tiab] OR Guinea[tiab] OR Gambia[tiab] OR Guinea-Bissau[tiab] OR Honduras[tiab] OR Haiti[tiab] OR India[tiab] OR Iran[tiab] OR Jordan[tiab] OR Kenya[tiab] OR Kyrgyz[tiab] OR Cambodia[tiab] OR Kiribati[tiab] OR Lao[tiab] OR Lebanon[tiab] OR Liberia[tiab] OR “Sri Lanka” [tiab] OR Lesotho[tiab] OR Morocco[tiab] OR Madagascar[tiab] OR Mali[tiab] OR Myanmar[tiab] OR Mongolia[tiab] OR Mozambique[tiab] OR Mauritania[tiab] OR Malawi[tiab] OR Niger[tiab] OR Nigeria[tiab] OR Nicaragua[tiab] OR Nepal[tiab] OR Pakistan[tiab] OR Philippines[tiab] OR “Papua New Guinea” [tiab] OR Korea[tiab] OR Rwanda[tiab] OR Sudan[tiab] OR Senegal[tiab] OR “Solomon Islands”[tiab] OR “Sierra Leone” [tiab] OR Somalia[tiab] OR Sudan[tiab] OR “São Tomé and Príncipe” [tiab] OR Eswatini[tiab] OR “Syrian Arab Republic” [tiab] OR Chad[tiab] OR Togo[tiab] OR Tajikistan[tiab] OR Timor-Leste[tiab] OR Tunisia[tiab] OR Tanzania[tiab] OR Uganda[tiab] OR Ukraine[tiab] OR Uzbekistan[tiab] OR Vietnam[tiab] OR Vanuatu[tiab] OR Samoa[tiab] OR Yemen[tiab] OR Zambia[tiab] OR Zimbabwe[tiab])) AND (pediatric[tiab] OR paediatric[tiab] OR infant[tiab] OR children[tiab] OR child[tiab] OR youth[tiab] OR childhood[tiab] OR routine[tiab] OR “life course”[tiab]).

### Appendix A.2. Web of Science (n = 46)

Afghanistan OR Angola OR Burundi OR Benin OR Burkina Faso OR Bangladesh OR Bolivia OR Bhutan OR Central African Republic OR “Côte d’Ivoire” OR Cameroon OR “Democratic Republic of Congo” OR Congo OR Comoros OR “Cabo Verde” OR Djibouti OR Algeria OR Egypt OR Eritrea OR Ethiopia OR Micronesia OR Ghana OR Guinea OR Gambia OR Guinea-Bissau OR Honduras OR Haiti OR India OR Iran OR Jordan OR Kenya OR Kyrgyz OR Cambodia OR Kiribati OR Lao OR Lebanon OR Liberia OR “Sri Lanka” OR Lesotho OR Morocco OR Madagascar OR Mali OR Myanmar OR Mongolia OR Mozambique OR Mauritania OR Malawi OR Niger OR Nigeria OR Nicaragua OR Nepal OR Pakistan OR Philippines OR “Papua New Guinea” OR Korea OR Rwanda OR Sudan OR Senegal OR Solomon Islands OR “Sierra Leone” OR Somalia OR Sudan OR “São Tomé and Príncipe” OR Eswatini OR “Syrian Arab Republic” OR Chad OR Togo OR Tajikistan OR Timor-Leste OR Tunisia OR Tanzania OR Uganda OR Ukraine OR Uzbekistan OR Vietnam OR Vanuatu OR Samoa OR Yemen OR Zambia OR Zimbabwe (Topic) and Pharmacy OR pharmacies OR pharmacist OR pharmacists OR chemist OR chemists OR druggist OR druggist (Topic) and vaccine OR vaccinat* OR immuniz* OR immunis* or inocula* (Topic) and pediatric OR paediatric OR infant OR children OR child OR youth OR childhood OR routine OR “life course” (Topic)|41 results.

### Appendix A.3. CINAHL (n = 10)

XB (Pharmacy OR pharmacies OR pharmacist OR pharmacists OR chemist OR chemists OR druggist OR druggists) AND XB (vaccine OR vaccinat* OR immuniz* OR immunis* or inocula*) AND XB (Afghanistan OR Angola OR Burundi OR Benin OR “Burkina Faso” OR Bangladesh OR Bolivia OR Bhutan OR “Central African Republic” OR “Côte d’Ivoire” OR Cameroon OR “Democratic Republic of Congo” OR Congo OR Comoros OR “Cabo Verde” OR Djibouti OR Algeria OR Egypt OR Eritrea OR Ethiopia OR Micronesia OR Ghana OR Guinea OR Gambia OR Guinea-Bissau OR Honduras OR Haiti OR India OR Iran OR Jordan OR Kenya OR Kyrgyz OR Cambodia OR Kiribati OR Lao OR Lebanon OR Liberia OR “Sri Lanka” OR Lesotho OR Morocco OR Madagascar OR Mali OR Myanmar OR Mongolia OR Mozambique OR Mauritania OR Malawi OR Niger OR Nigeria OR Nicaragua OR Nepal OR Pakistan OR Philippines OR “Papua New Guinea” OR Korea OR Rwanda OR Sudan OR Senegal OR “Solomon Islands” OR “Sierra Leone” OR Somalia OR Sudan OR “São Tomé and Príncipe” OR Eswatini OR “Syrian Arab Republic” OR Chad OR Togo OR Tajikistan OR Timor-Leste OR Tunisia OR Tanzania OR Uganda OR Ukraine OR Uzbekistan OR Vietnam OR Vanuatu OR Samoa OR Yemen OR Zambia OR Zimbabwe) AND XB (pediatric OR paediatric OR infant OR children OR child OR youth OR childhood OR routine OR “life course”).

### Appendix A.4. Embase (n = 19)

(pharmacy:ti,ab,kw OR pharmacies:ti,ab,kw OR pharmacist:ti,ab,kw OR pharmacists:ti,ab,kw OR chemist:ti,ab,kw OR chemists:ti,ab,kw OR druggist:ti,ab,kw OR druggists:ti,ab,kw) AND (vaccine:ti,ab,kw OR vaccines:ti,ab,kw OR vaccinate:ti,ab,kw OR vaccinator:ti,ab,kw OR vaccination:ti,ab,kw OR vaccinations:ti,ab,kw OR immunize:ti,ab,kw OR immunizes:ti,ab,kw OR immunizer:ti,ab,kw OR immunization:ti,ab,kw OR immunizations:ti,ab,kw OR immunise:ti,ab,kw OR immunises:ti,ab,kw OR immunisers:ti,ab,kw OR immuniser:ti,ab,kw OR immunisation:ti,ab,kw OR inoculate:ti,ab,kw OR inoculates:ti,ab,kw OR inoculation:ti,ab,kw OR inoculations:ti,ab,kw OR inoculator:ti,ab,kw OR inoculators:ti,ab,kw) AND ((afghanistan:ti,ab,kw OR angola:ti,ab,kw OR burundi:ti,ab,kw OR benin:ti,ab,kw OR ‘burkina faso’:ti,ab,kw OR bangladesh:ti,ab,kw OR bolivia:ti,ab,kw OR bhutan:ti,ab,kw OR ‘central african republic’:ti,ab,kw OR ‘cote d`ivoire’:ti,ab,kw OR cameroon:ti,ab,kw OR ‘democratic republic of congo’:ti,ab,kw OR congo:ti,ab,kw OR comoros:ti,ab,kw OR ‘cabo verde’:ti,ab,kw OR djibouti:ti,ab,kw OR algeria:ti,ab,kw OR egypt:ti,ab,kw OR eritrea:ti,ab,kw OR ethiopia:ti,ab,kw OR micronesia:ti,ab,kw OR ghana:ti,ab,kw OR guinea:ti,ab,kw OR gambia:ti,ab,kw OR ‘guinea bissau’:ti,ab,kw OR honduras:ti,ab,kw OR haiti:ti,ab,kw OR india:ti,ab,kw OR iran:ti,ab,kw OR jordan:ti,ab,kw OR kenya:ti,ab,kw OR kyrgyz:ti,ab,kw OR cambodia:ti,ab,kw OR kiribati:ti,ab,kw OR lao:ti,ab,kw OR lebanon:ti,ab,kw OR liberia:ti,ab,kw OR ‘sri lanka’:ti,ab,kw OR lesotho:ti,ab,kw OR morocco:ti,ab,kw OR madagascar:ti,ab,kw OR mali:ti,ab,kw OR myanmar:ti,ab,kw OR mongolia:ti,ab,kw OR mozambique:ti,ab,kw OR mauritania:ti,ab,kw OR malawi:ti,ab,kw OR niger:ti,ab,kw OR nigeria:ti,ab,kw OR nicaragua:ti,ab,kw OR nepal:ti,ab,kw OR pakistan:ti,ab,kw OR philippines:ti,ab,kw OR ‘papua new guinea’:ti,ab,kw OR korea:ti,ab,kw OR rwanda:ti,ab,kw OR senegal:ti,ab,kw OR ‘solomon islands’:ti,ab,kw OR ‘sierra leone’:ti,ab,kw OR somalia:ti,ab,kw OR sudan:ti,ab,kw OR ‘são tomé’:ti,ab,kw) AND príncipe:ti,ab,kw OR eswatini:ti,ab,kw OR ‘syrian arab republic’:ti,ab,kw OR chad:ti,ab,kw OR togo:ti,ab,kw OR tajikistan:ti,ab,kw OR ‘timor leste’:ti,ab,kw OR tunisia:ti,ab,kw OR tanzania:ti,ab,kw OR uganda:ti,ab,kw OR ukraine:ti,ab,kw OR uzbekistan:ti,ab,kw OR vietnam:ti,ab,kw OR vanuatu:ti,ab,kw OR samoa:ti,ab,kw OR yemen:ti,ab,kw OR zambia:ti,ab,kw OR zimbabwe:ti,ab,kw) AND (pediatric:ti,ab,kw OR paediatric:ti,ab,kw OR infant:ti,ab,kw OR children:ti,ab,kw OR child:ti,ab,kw OR youth:ti,ab,kw OR childhood:ti,ab,kw OR routine:ti,ab,kw OR ‘life course’:ti,ab,kw).

### Appendix A.5. Cochrane (n = 24)

Pharmacy OR pharmacies OR pharmacist OR pharmacists OR chemist OR chemists OR druggist OR druggists in Title Abstract Keyword AND vaccine OR vaccines OR vaccinate OR vaccinator OR vaccination OR vaccinations OR immunize OR immunizes OR immunizer OR immunization OR immunizations OR immunise OR immunises OR immunisers OR immuniser OR immunisation OR inoculate OR inoculates OR inoculation OR inoculations OR inoculator OR inoculators in Title Abstract Keyword AND Afghanistan OR Angola OR Burundi OR Benin OR Burkina Faso OR Bangladesh OR Bolivia OR Bhutan OR Central African Republic OR ‘Cote d`Ivoire’ OR Cameroon OR Democratic Republic of Congo OR Congo OR Comoros OR Cabo Verde OR Djibouti OR Algeria OR Egypt OR Eritrea OR Ethiopia OR Micronesia OR Ghana OR Guinea OR Gambia OR Guinea-Bissau OR Honduras OR Haiti OR India OR Iran OR Jordan OR Kenya OR Kyrgyz OR Cambodia OR Kiribati OR Lao OR Lebanon OR Liberia OR Sri Lanka OR Lesotho OR Morocco OR Madagascar OR Mali OR Myanmar OR Mongolia OR Mozambique OR Mauritania OR Malawi OR Niger OR Nigeria OR Nicaragua OR Nepal OR Pakistan OR Philippines OR Papua New Guinea OR Korea OR Rwanda OR Sudan OR Senegal OR Solomon Islands OR Sierra Leone OR Somalia OR Sudan OR São Tomé and Príncipe OR Eswatini OR Syrian Arab Republic OR Chad OR Togo OR Tajikistan OR Timor-Leste OR Tunisia OR Tanzania OR Uganda OR Ukraine OR Uzbekistan OR Vietnam OR Vanuatu OR Samoa OR Yemen OR Zambia OR Zimbabwe in Title Abstract Keyword AND pediatric OR paediatric OR infant OR children OR child OR youth OR childhood OR routine OR “life course” in Title Abstract Keyword—(Word variations have been searched).

## Appendix B. Excluded Full Text Studies

**Table A1:** Studies identified for full-text review but excluded from the study.

Author and Year	Reason for Exclusion
Ade-adekunle and colleagues 2024 [20]	Article type (commentary)
Aderemi-Williams and Igwilo 2007 [33]	Does not include paediatric patients
Chukhray and colleagues 2023 [34]	Does not include pharmacists in immunisation administration
Isah and Ubaka 2021 [35]	Does not include paediatric patients
Jalloh and colleagues 2019 [36]	Does not include pharmacists in immunisation administration
Khan and colleagues 2020 [23]	Article type (commentary)
Kiringa 2012 [37]	Article type (conference abstract)
Sebastian and colleagues 2018 [21]	Article type (commentary)

## References

[1] World Health Organization Vaccines: The Powerful Innovations Bringing WHO’s Mission to Life Every Day. https://www.who.int/news-room/commentaries/detail/vaccines-the-powerful-innovations-bringing-who-s-mission-to-life-every-day.

[2] World Health Organization Vaccines and Immunization. https://www.who.int/health-topics/vaccines-and-immunization#tab=tab_1.

[3] UNICEF COVID-19 Pandemic Leads to Major Backsliding on Childhood Vaccinations, New WHO, UNICEF Data Shows. https://www.unicef.org/press-releases/covid-19-pandemic-leads-major-backsliding-childhood-vaccinations-new-who-unicef-data.

[4] World Health Organization Immunization Coverage. https://www.who.int/en/news-room/fact-sheets/detail/immunization-coverage.

[5] Gavi The Zero-Dose Child: Explained. https://www.gavi.org/vaccineswork/zero-dose-child-explained.

[6] World Health Organization WHO/UNICEF Immunization Coverage Estimates 2024. https://cdn.who.int/media/docs/default-source/immunization/immunization-coverage/wuenic_notes.pdf?sfvrsn=88ff590d_23&download=true.

[7] Hogan D., Gupta A. (2023). Why Reaching Zero-Dose Children Holds the Key to Achieving the Sustainable Development Goals. Vaccines.

[8] Gavi The Gavi Investment Opportunity 2021–2025. https://www.gavi.org/gavi-investment-opportunity-2021-2025.

[9] Cata-Preta B.O., Santos T.M., Wendt A., Hogan D.R., Mengistu T., Barros A.J.D., Victora C.G. (2022). Ethnic disparities in immunisation: Analyses of zero-dose prevalence in 64 countries. BMJ Glob. Health.

[10] Santos T.M., Cata-Preta B.O., Wendt A., Arroyave L., Hogan D.R., Mengistu T., Barros A.J.D., Victora C.G. (2022). Religious Affiliation as a Driver of Immunization Coverage: Analyses of Zero-Dose Vaccine Prevalence in 66 Low- and Middle-Income Countries. Front. Public Health.

[11] Wendt A., Santos T.M., Cata-Preta B.O., Costa J.C., Mengistu T., Hogan D.R., Victora C.G., Barros A.J. (2022). Children of More Empowered Women Are Less Likely to Be Left without Vaccination in Low-and Middle-Income Countries: A Global Analysis of 50 DHS Surveys. J. Glob. Health.

[12] World Health Organization (2022). Ensuring the Integration of Refugees and Migrants in Immunization Policies, Planning and Service Delivery Globally.

[13] World Health Organization Immunization Agenda 2030: A Global Strategy to Leave No One Behind. https://www.who.int/teams/immunization-vaccines-and-biologicals/strategies/ia2030.

[14] Romero-Mancilla M.S., Mora-Vargas J., Ruiz A. (2023). Pharmacy-based immunization: A systematic review. Front. Public Health.

[15] IQVIA Trends in Vaccine Administration in the United States. https://www.iqvia.com/insights/the-iqvia-institute/reports-and-publications/reports/trends-in-vaccine-administration-in-the-united-states.

[16] Kirkdale C.L., Nebout G., Megerlin F., Thornley T. (2017). Benefits of pharmacist-led flu vaccination services in community pharmacy. Ann. Pharm. Fr..

[17] Pharmacy Guild of Australia COVID-19 Vaccinations Delivered Through Community Pharmacies Hit 9 Million. https://www.guild.org.au/news-events/news/2022/covid-19-vaccinations-delivered-through-community-pharmacies-hit-9-million.

[18] Yemeke T.T., Mitgang E., Wedlock P.T., Higgins C., Chen H.H., Pallas S.W., Abimbola T., Wallace A., Bartsch S.M., Lee B.Y. (2021). Promoting, seeking, and reaching vaccination services: A systematic review of costs to immunization programs, beneficiaries, and caregivers. Vaccine.

[19] Ayenew W., Anagaw Y.K., Limenh L.W., Simegn W., Bizuneh G.K., Bitew T., Minwagaw T., Fitigu A.E., Dessie M.G., Asmamaw G. (2024). Readiness of and barriers for community pharmacy professionals in providing and implementing vaccination services. BMC Health Serv. Res..

[20] Ade-adekunle O.A., Adekunle F.O., Idris Z.O., Babalola D.G. (2024). Enhancing diphtheria vaccination efficacy in Nigeria: Integrating community pharmacists for improved accessibility, distribution, and awareness. Clin. Epidemiol. Glob. Health.

[21] Sebastian J., Parthasarathi G., Ravi M.D. (2018). Is there a Role for Pharmacist in Safety Monitoring of Vaccines?. Indian J. Pharm. Educ. Res..

[22] G/Michael Beyene K., Tamiru M.T. (2025). Quality and safety requirements for pharmacy-based vaccination in resource-limited countries. Trop. Med. Health.

[23] Khan A., Bibi A., Sheraz Khan K., Raza Butt A., Alvi H.A., Zahra Naqvi A., Mushtaq S., Khan Y.H., Ahmad N. (2020). Routine Pediatric Vaccination in Pakistan During COVID-19: How Can Healthcare Professionals Help?. Front. Pediatr..

[24] Higgins J.P.T., Thomas J., Chandler J., Cumpston M., Li T., Page M.J., Welch V.A. (2019). Cochrane Handbook for Systematic Reviews of Interventions.

[25] Moher D., Shamseer L., Clarke M., Ghersi D., Liberati A., Petticrew M., Shekelle P., Stewart L.A., PRISMA-P Group (2015). Preferred reporting items for systematic review and meta-analysis protocols (PRISMA-P) 2015 statement. Syst. Rev..

[26] The World Bank World Bank Country and Lending Groups. https://datahelpdesk.worldbank.org/knowledgebase/articles/906519-world-bank-country-and-lending-groups.

[27] Koehler T., Brown A. (2017). A global picture of pharmacy technician and other pharmacy support workforce cadres. Res. Soc. Adm. Pharm..

[28] Arvind R.K., Beerwala F.A., Wali S.C., Parihar A.S., Ganachari M.S., Bhandari R. (2022). A Randomized, Single Centered, Parallel and Open Labelled Interventional Study on Effectiveness of Clinical Pharmacists on Adverse Event Following Immunization (AEFI) in Pediatric Population. Curr. Drug Saf..

[29] Ayele A.A., Cosh S., Islam M.S., East L. (2022). Role of community pharmacy professionals in child health service provision in Ethiopia: A cross-sectional survey in six cities of Amhara regional state. BMC Health Serv. Res..

[30] Batarseh Y.S., Darwish Elhajji F.W., Shammas S., Darwish R., Fakhoury R., Haj Ahmad M.A., Al-Rusasi A., Jarrar L. (2021). Perception and attitude of the public on vaccine practices and pharmacists as immunizers in Jordan. J. Pharm. Health Serv. Res..

[31] Shawon M.S.R., Adhikary G., Ali M.W., Shamsuzzaman M., Ahmed S., Alam N., Shackelford K.A., Woldeab A., Lim S.S., Levine A. (2018). General service and child immunization-specific readiness assessment of healthcare facilities in two selected divisions in Bangladesh. BMC Health Serv. Res..

[32] Landis J.R., Koch G.G. (1977). The measurement of observer agreement for categorical data. Biometrics.

[33] Aderemi-Williams R.I., Igwilo C.I. (2007). Community Pharmacies as Possible Centres for Routine Immunization. Niger. Q. J. Hosp. Med..

[34] Chukhray I., Levytska O., Oliinyk P., Hadiak I., Bilushchak H. (2023). Social-pharmaceutical aspects of parents’ attitudes towards children’s vaccination. ScienceRise Pharm. Sci..

[35] Isah A., Ubaka C.M. (2021). Pharmacists’ readiness to receive, recommend and administer COVID-19 vaccines in an African country: An online multiple-practice settings survey in Nigeria. Malawi Med. J..

[36] Jalloh M.F., Bennett S.D., Alam D., Kouta P., Lourenço D., Alamgir M., Feldstein L.R., Ehlman D.C., Abad N., Kapil N. (2019). Rapid behavioral assessment of barriers and opportunities to improve vaccination coverage among displaced Rohingyas in Bangladesh, January 2018. Vaccine.

[37] Kiringa G. (2012). Updates from a randomised, double blind placebo controlled phase IIb tuberculosis vaccine trial in HIV-negative infants in Western Kenya. Trop. Med. Int. Health.

[38] Indian Pharmaceutical Association Request Letter for Training Partner to Train Pharmacists as Vaccinators. https://ipapharma.org/wp-content/uploads/2022/06/Request-letter-for-training-partner-to-train-pharmacists-as-vaccinators-for-Website.pdf.

[39] Times of India Pharmacists to Become Vaccinators, 12.5 lakh to Get Training from May. https://timesofindia.indiatimes.com/india/pharmacists-to-become-vaccinators-12-5-lakh-to-get-training-from-may/articleshow/97234192.cms.

[40] International Pharmaceutical Federation (FIP) Supporting Life-Course Immunisation Through Pharmacy-Based Vaccination: Enabling Equity, Access and Sustainability A Toolkit for Pharmacists. https://www.fip.org/file/5588.

[41] World Health Organization Service Availability and Readiness Assessment (SARA). https://www.who.int/data/data-collection-tools/service-availability-and-readiness-assessment-(sara).

[42] Meyer H., Khosa L.A., Nhira S., Chiloane L., Sibanda M., Schönfeldt M., Burnett R.J. (2018). Childhood vaccination and the role of the pharmacist. S. Afr. Pharm. J..

[43] Agah S., Nsofor I., Zeng W. (2026). Evaluating a Behavioral Insights-Informed Social Media Campaign to Increase HPV Vaccination During Routine Immunizations in Nigeria. Vaccines.

[44] Alnahar S.A., Gkountouras G., Darwish R.M., Bates I. (2022). Community pharmacists workforce readiness to deliver vaccination services: A cross-sectional study from Jordan. Pharmacol. Res. Perspect..

[45] Mukattash T.L., Jarab A.S., Abu Farha R.K., Nusair M.B., Muqatash S.A. (2021). Pharmacists’ perspectives on providing the COVID-19 vaccine in community pharmacies. J. Pharm. Health Serv. Res..

[46] Mayora C., Kitutu F.E., Kandala N.B., Ekirapa-Kiracho E., Peterson S.S., Wamani H. (2018). Private retail drug shops: What they are, how they operate, and implications for health care delivery in rural Uganda. BMC Health Serv. Res..

[47] Aelita K., Roksolana K., Liliia B., Oksana L. (2022). Vaccine Prevention of Tuberculosis in Children: Outstanding Issues Family Medicine Systems in Ukraine. Arch. Pharm. Pract..

[48] McKeirnan K.C., Truter I., Fogarty T.L. (2024). A systematic review of immunization administration training for African pharmacists and student pharmacists. Am. J. Pharm. Educ..

[49] El Hajj M.S., Saleh M., Ibrahim N., El-Awaisi A., Baraka M., Stewart D., Nasr Z.G. (2024). A cross-sectional study of vaccination-related education in pharmacy programs in the Middle East. Am. J. Pharm. Educ..

[50] Oladigbolu A.A., Okafor U.G., Oluwaseyi C.O., Ashore O.M. (2025). Community pharmacy workforce willingness, readiness, and infrastructural capacity to deliver vaccination services: A cross-sectional survey in Nigeria. BMC Health Serv. Res..

[51] Valeiro C., Silva V., Balteiro J., Patterson D., Bezerra G., Mealiff K., Matos C., Jesus Â., Joaquim J. (2025). Pharmacy technicians in immunization services: Mapping roles and responsibilities through a scoping review. Healthcare.

[52] McKeirnan K., Sarchet G. (2019). Implementing immunizing pharmacy technicians in a federal healthcare facility. Pharmacy.

[53] McKeirnan K.C., Frazier K.R., Nguyen M., MacLean L.G. (2018). Training pharmacy technicians to administer immunizations. J. Am. Pharm. Assoc..

[54] DeMarco M., Carter C., Houle S.K.D., Waite N.M. (2022). The role of pharmacy technicians in vaccination services: A scoping review. J. Am. Pharm. Assoc..

[55] Dimitris M.C., Gittings M., King N.B. (2021). How global is global health research? A large-scale analysis of trends in authorship. BMJ Glob. Health.

